# Complete chromosome-level genome assembly data from the tawny crazy ant, *Nylanderia fulva* (Mayr) (Hymenoptera: Formicidae)

**DOI:** 10.1016/j.dib.2022.108833

**Published:** 2022-12-16

**Authors:** Margaret L. Allen, Perot Saelao, Godfrey P. Miles, David C. Cross, JoVonn G. Hill, Edward L. Vargo, Michael J. Grodowitz

**Affiliations:** a US Department of Agriculture, Agricultural Research Service, Southeast Area, Biological Control of Pests Research Unit, National Biological Control Laboratory, 59 Lee Road, Stoneville, MS 38776, United States; b US Department of Agriculture, Agricultural Research Service, Plains Area, Veterinary Pest Genetics Research Unit, US Livestock Insects Research Lab, 2700 Fredericksburg Road, Kerrville, TX 78028, United States; c Mississippi State University, Department of Biochemistry, Molecular Biology, Entomology and Plant Pathology, 100 Old Hwy. 12, Mississippi State, MS 39762, United States; d Department of Entomology, Texas A&M University, 2143 TAMU, College Station, TX 77843, United States

**Keywords:** Invasive ant, Eusocial insect, Synteny, Updated genome, Revised assembly

## Abstract

The tawny crazy ant, *Nylanderia fulva* (Mayr) (Hymenoptera: Formicidae) has a native range that extends from northern Argentina to southern Brazil. In the U.S.A. this species has often been misidentified as *Nylanderia* (*Paratrechina*) *pubens* or *N. cf. pubens* and has likely been present in Florida and Texas for several decades [1]. In the early 2000’s explosive population growth in Texas and neighboring states drew renewed taxonomic focus. Genetic analyses [2,3] aided in identifying the pest species as *N. fulva*. This species poses an invasive threat to native flora and fauna and human structures. In its invasive range it has been reported to displace another invasive species, the red imported fire ant. The specimens used for genome sequencing were obtained from the coastal region of Mississippi. DNA was extracted from pupae. The genome data set was deposited to the National Center for Biotechnology Information as submission ID: SUB10775679, Project ID: PRJNA796544, Accession IDs: SAMN24895442 and JAKFQQ000000000. The organism taxid is 613905, locus tag prefixes are L1K79. The assembly, USDA_Nfulva_1.0, was generated in collaboration with Dovetail Genomics (now Cantata Bio) to yield a chromosome-level assembly of 375 Mb with a 15.67 Mb N50 and 78X coverage and revealing 16 putative chromosomes. This high-quality chromosome-level genome assembly was released prior to publication as a public service to the research community.

**Specifications Table**

**Table:** 

Subject	Entomology and Insect Science, Phylogeny and Evolution
Specific subject area	Myrmecology, Invasive species, Social insects, Hymenoptera, Formicidae, Chromosomes
Type of data	Scaffolded genome assemblies from DNA sequencing of worker pupae (sterile females), GenBank identification numbers, Tables describing summary statistics of the assemblies, topologically associated domains (TADs) statistics, and listing the chromosomes with links to publicly deposited assemblies. A Figure illustrating genome scaffolding improvement showing contact mapping.
How the data were acquired	DNA samples were extracted from ant pupae and used to prepare chromosome-level sequencing libraries. Libraries were then processed by high-throughput sequencing. HiRise scaffolding was done using Dovetail scaffolding assembly tools and analysis reports were provided by Dovetail Genomics (now Cantata Bio).
Data format	Raw Analyzed
Description of data collection	Field-collected ants were identified by the Mississippi Entomological Museum, separated by developmental stage and flash frozen in liquid nitrogen. Samples were mailed in dry ice to Dovetail Genomics (now Cantata Bio) for Omni-C library preparation, sequencing, and scaffold assembly. Sequences were aligned to the *Nylanderia fulva* (Taxonomy ID 613905) reference genome assembly GCA_005281655.1 [4] using bwa (https://github.com/lh3/bwa). The separations of Dovetail Omni-C read pairs mapped within draft scaffolds were analyzed by HiRise to produce a likelihood model for genomic distance between read pairs, and the model was used to identify and break putative misjoins, to score prospective joins, and make joins above confidence threshold [5], [6], [7].
Data source location	• Institutions: Mississippi State University, USDA Agricultural Research Service • City/Town/Region: Ocean Springs, Mississippi • Country: USA
Data accessibility	Raw sequence reads and chromosome-level assemblies can be obtained through NCBI Project accession number PRJA796544 https://www.ncbi.nlm.nih.gov/bioproject/PRJNA796544, assembly number GCA_024268025.1 https://www.ncbi.nlm.nih.gov/data-hub/genome/GCA_024268025.1. The whole genome master sequence accession is JAKFQQ000000000.1 https://www.ncbi.nlm.nih.gov/nuccore/JAKFQQ000000000.1 (also https://www.ncbi.nlm.nih.gov/nuccore/2271424038). Chromosome assembly accession numbers and links are provided in Table 3.

## Value of the Data

• This is a high-quality chromosome-level genome of the invasive tawny crazy ant, *Nylanderia fulva* (TCA), an important pest ant in its introduced regions.
• Researchers studying ant and social insect genomics, comparative genomics, and evolution will find value in the chromosome-level genome, particularly for studying supergenes and synteny.
• Ants (family Formicidae, order Hymenoptera) have extremely diverse chromosome-level genomes, with haploid chromosome number varying between 1 and 60 [8]. These data will help study this phenomenon.
• The dataset can be used to study genes involved in sexual and social phenotypic and behavioral polymorphisms.
• The dataset will help clarify the evolution of chemical communication and venom genes and biosynthetic pathways.

## 1 Objective

Of the approximately 14,000 extant ant species described, many have had their genomes (https://www.antweb.org, accessed July 2022) and transcriptomes sequenced and annotated [4,^9^,10]. While many sequencing efforts have focused on pest ant species from around the world, chromosome-level sequencing and annotation of ant species is far from complete. The main objective was to improve the public genome assembly to chromosome level to facilitate evolutionary and functional analyses of ants and other social insects.

## 2 Data Description

**Fig. 1:**
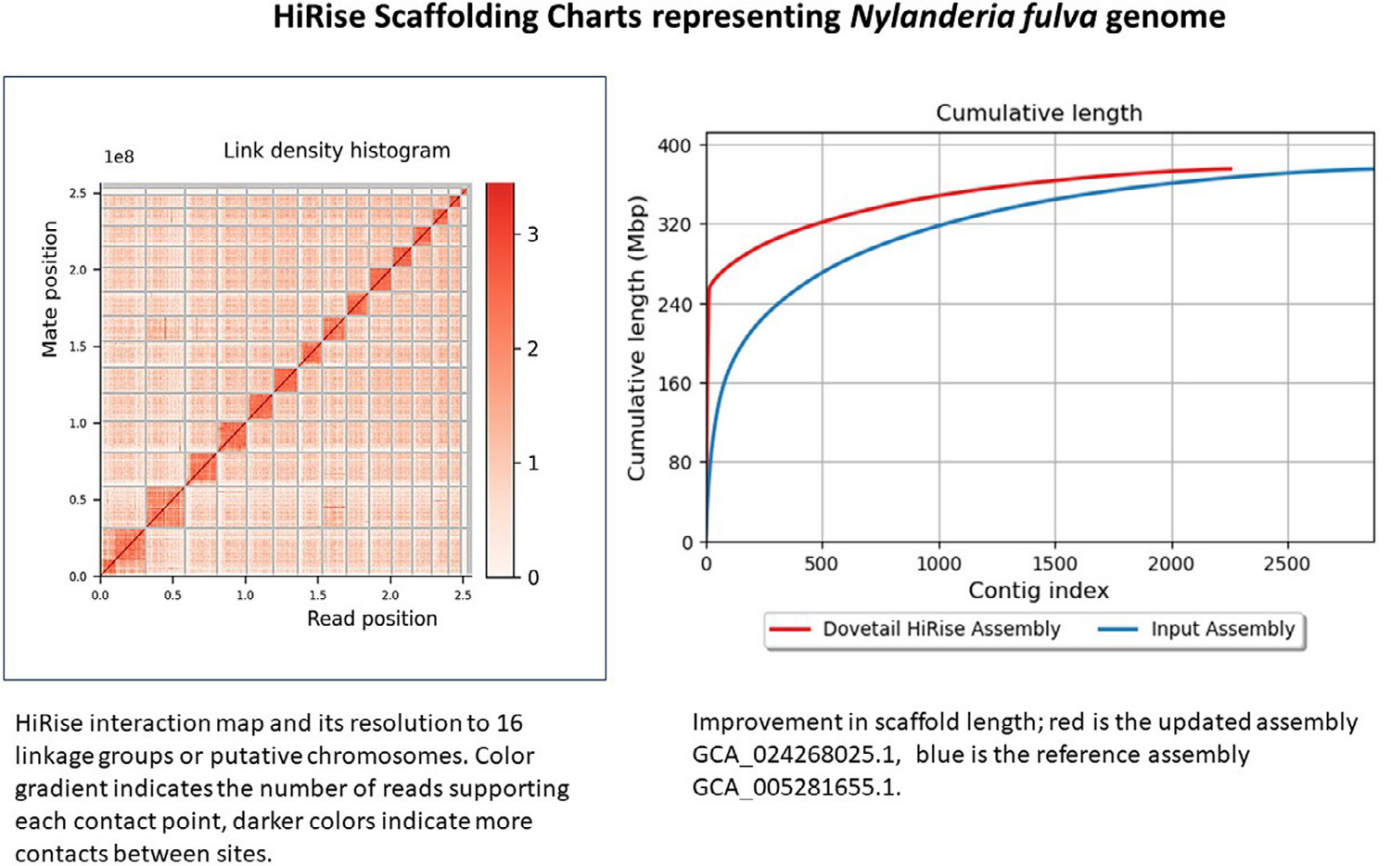
Improved assembly of the *Nylanderia fulva* genome illustrated by density histogram showing distinct chromosome separations. Cumulative length chart illustrates improvement of contigs compared to the input reference genome.

The genome data set was deposited to the National Center for Biotechnology Information (NCBI) as submission ID: SUB10775679, Project ID: PRJNA796544, Accession IDs: SAMN24895442 and JAKFQQ000000000. The organism taxonomy ID is 613905, and locus tag prefixes are L1K79. The assembly, USDA_Nfulva_1.0, was generated in collaboration with Dovetail Genomics (now Cantata Bio) to yield a chromosome assembly of 375 Mb with a 15.67 Mb N50 and 15.9 Kb N90, at 78X coverage (Table 1), and revealing 16 putative chromosomes (Table 3). Scaffolding improvements to the reference genome GCA_005281655.1 are illustrated in Fig. 1 and Table 2.

**Table 1:** Summary statistics comparison of *Nylanderia fulva* genome assemblies.

Assembly	Total Length (bp)	N50	L50	N90	L90	Scaffolds	Longest Scaffold	BUSCO*
Input Assembly	375,107,333	443,094	131	48,202	1,345	2869	5,845,509	241 (94.51%)
Dovetail HiRise Assembly	375,170,133	15,670,585	10	51,923	767	2255	31,358,142	241 (94.51%)

⁎ BUSCO metrics for the chromosome-level assembly were nearly identical to those for the input assembly. Complete(C) BUSCOs 241 (94.51%, shown in Table), complete and single copy (S) 234 (91.76%), complete and duplicated (D) 7, fragmented (F) 9 in the input assembly and 8 in the chromosome-level assembly, missing (M) 5 in the input assembly and 6 in the chromosome-level assembly, total groups searched 255. The BUSCO version used was 4.05 with lineage dataset eukaryota_odb 10 [11].

**Table 2:** Topologically associating domains (TAD) statistics at different resolutions. 46.68% of the genome was contained in 29 A compartments, representing euchromatin. 49.42% of the genome was contained in 30 B compartments, representing heterochromatin.

Resolution (kbp)	Number of TADs	Mean TAD Size (bp)	Basepairs in TADs (kbp)	Percent genome contained in TADs
10	176	319,715	522	20.50%
25	86	533,430	394	15.47%
50	50	980,000	460	18.02%

**Table 3:** *Nylanderia fulva* Chromosome sizes, accession numbers and links.

Contig	Size	Accession	Link
Chromosome/Scaffold_1	31.4 Mb	CM044288.1	https://www.ncbi.nlm.nih.gov/nuccore/CM044288.1
Chromosome/Scaffold_2	27.2 Mb	CM044289.1	https://www.ncbi.nlm.nih.gov/nuccore/CM044289.1
Chromosome/Scaffold_3	21.9 Mb	CM044290.1	https://www.ncbi.nlm.nih.gov/nuccore/CM044290.1
Chromosome/Scaffold_4	20.3 Mb	CM044291.1	https://www.ncbi.nlm.nih.gov/nuccore/CM044291.1
Chromosome/Scaffold_5	18.4 Mb	CM044292.1	https://www.ncbi.nlm.nih.gov/nuccore/CM044292.1
Chromosome/Scaffold_6	17.0 Mb	CM044293.1	https://www.ncbi.nlm.nih.gov/nuccore/CM044293.1
Chromosome/Scaffold_7	17.0 Mb	CM044294.1	https://www.ncbi.nlm.nih.gov/nuccore/CM044294.1
Chromosome/Scaffold_8	16.6 Mb	CM044295.1	https://www.ncbi.nlm.nih.gov/nuccore/CM044295.1
Chromosome/Scaffold_9	15.7 Mb	CM044296.1	https://www.ncbi.nlm.nih.gov/nuccore/CM044296.1
Chromosome/Scaffold_10	15.7 Mb	CM044297.1	https://www.ncbi.nlm.nih.gov/nuccore/CM044297.1
Chromosome/Scaffold_11	13.9 Mb	CM044298.1	https://www.ncbi.nlm.nih.gov/nuccore/CM044298.1
Chromosome/Scaffold_12	13.4 Mb	CM044299.1	https://www.ncbi.nlm.nih.gov/nuccore/CM044299.1
Chromosome/Scaffold_13	11.4 Mb	CM044300.1	https://www.ncbi.nlm.nih.gov/nuccore/CM044300.1
Chromosome/Scaffold_14	8.8 Mb	CM044301.1	https://www.ncbi.nlm.nih.gov/nuccore/CM044301.1
Chromosome/Scaffold_15	4.7 Mb	CM044302.1	https://www.ncbi.nlm.nih.gov/nuccore/CM044302.1
Chromosome/Scaffold_16	1.5 Mb	CM044303.1	https://www.ncbi.nlm.nih.gov/nuccore/CM044303.1

## 3 Experimental Design, Materials and Methods

### 3.1 Insect specimens

A portion of a TCA colony was collected in Ocean Springs, MS on May 7, 2021. Ants were found in a wooded area on the grounds of the Cedar Point facility of the Gulf Coast Research Laboratory (30.39° N, 88.78° W) that has been known to have TCA for at least eight years. The colony had several queens and an abundance of brood. It was in a rotting limb of a small understory tree. Portions of the limb were broken off and TCA were shaken into a collection box for transport to Mississippi State University, Starkville, MS. The colony was maintained in the lab for over 45 days. A fluon (Insect-a-slip, BioQuip) film applied inside the lower rim of the holding boxes kept the ants contained. The ants and their brood were manually transferred with small hobby brushes to separate clean containers. Using a Leica M125 stereo microscope (8-100X), over 200mg of both early pupae and mixed age larvae were placed in corresponding cryotubes. The tubes were chilled on ice for five minutes and then dropped into liquid nitrogen for at least four minutes to flash freeze the tissue. An aluminum tube block pre-chilled to -75°C was used to transfer the tube to an ultra-cold freezer at the same temperature. The samples were shipped on dry ice to the Dovetail Genomics lab for sequencing and subsequent analyses.

### 3.2 DNA preparation and sequencing

DNA samples were quantified using Qubit 2.0 Fluorometer (Life Technologies, Carlsbad, CA, USA). For Dovetail Omni-C library, chromatin was fixed in place with formaldehyde in the nucleus. Fixed chromatin was digested with DNase I and then extracted, chromatin ends were repaired and ligated to a biotinylated bridge adapter followed by proximity ligation of adapter-containing ends. After proximity ligation, crosslinks were reversed, and the DNA purified. Purified DNA was treated to remove biotin that was not internal to ligated fragments. Sequencing libraries were generated using NEBNext Ultra enzymes and Illumina-compatible adapters. Biotin-containing fragments were isolated using streptavidin beads before PCR enrichment of library. The PacBio SMRTbell library (∼20kb) for PacBio Sequel was constructed using SMRTbell Express Template Prep Kit 2.0 (PacBio, Menlo Park, CA, USA) using the manufacturer recommended protocol. The library was bound to polymerase using the Sequel II Binding Kit 2.0 (PacBio) and loaded onto PacBio Sequel II. Sequencing was performed on PacBio Sequel II 8M SMRT cells. Scaffolding was done using Illumina HiSeqX platform, paired-end sequencing, and Dovetail HiRise scaffolding assembly tools were provided by Dovetail Genomics (now Cantata Bio).

### 3.3 Data processing analysis

The input *de novo* assembly and Dovetail OmniC library reads were used as input data for HiRise, a software pipeline designed specifically for using proximity ligation data to scaffold genome assemblies [6]. Dovetail OmniC library sequences were aligned to the draft input assembly using bwa (https://github.com/lh3/bwa). The separations of Dovetail OmniC read pairs mapped within draft scaffolds were analyzed by HiRise to produce a likelihood model for genomic distance between read pairs, and the model was used to identify and break putative misjoins, to score prospective joins, and make joins above a threshold. 628 joins were made by HiRise, and 14 breaks were made to the input assembly.

Hi-C contact matrices were generated in two formats: cool and hic. Both contact matrices were generated from the same BAM file by using read pairs where both ends were aligned with a mapping quality of 60. Topologically associated domains (TADs) are fundamental units of chromatin topology, wherein all the chromatin is in close physical proximity. It is thought that regulatory signals can be conveyed more easily within a TAD than between TADs. TAD boundaries often occur at CTCF binding sites and are thought to be established and maintained by Cohesin/CTCF complex. TADs were identified using the Arrowhead program implemented in the Juicertools package. TADs were called at three different resolutions: 10 kbp, 25 kbp, and 50 kbp. The parameters used were -k KR -m 2000 -r 10000, -k KR -m 2000 -r 25000, and -k KR -m 2000 -r 50000. A/B compartments were identified at 1 Mbp using the eigenvector program implemented in the JuicerTools package (https://github.com/aidenlab/ JuicerTools). The parameters used were KR BP 1000000. TAD statistics are presented in Table 2. Isochores, extended genomic regions (typically 300kb to multimegabase) of uniform, characteristic GC content, were predicted using the isofinder program; none were predicted. The parameters used were 0.90 p2 3000. The output was post-processed and converted to BEDPE format. 959 CTCF sites were predicted using the CREAD program. The position weight matrix was downloaded from CTCFBSDB 2.0 website. The output was then post-processed to convert it to a bed file. Multires files were generated using the clodius package. Scaffolding and TAD reports were provided by Dovetail.

## Ethics Statements

The authors state that the work described here does not include human nor animal studies. The authors have no conflicts of interest. The work has not been published previously and it is not under consideration for publication elsewhere; its publication is approved by all authors and tacitly or explicitly by the responsible authorities where the work was carried out; and that, if accepted, it will not be published elsewhere in the same form, in English or in any other language, including electronically without the written consent of the copyright holder.

## CRediT Author Statement

**Margaret L. Allen:** Conceptualization, Supervision, Writing – Original draft preparation. Visualization, Investigation; **Perot Saelao:** Data curation, Software & Methodology Validation Writing – Reviewing and Editing; **Godfrey P. Miles:** Writing – Reviewing and Editing; **David C. Cross:** Sample Collection, Writing – Reviewing and Editing; **JoVonn G. Hill:** Supervision, Writing – Reviewing and Editing; **Edward L. Vargo:** Supervision, Data curation, Writing – Reviewing and Editing; **Michael J. Grodowitz:** Supervision, Writing – Reviewing and Editing.

## Declaration of Competing Interest

The authors declare that they have no known competing financial interests or personal relationships that could have appeared to influence the work reported in this paper.

## Data Availability

USDA_Nfulva_1.0 (Original data) (NCBI GenBank).
